# Stress distribution characteristics of overburden strata during thick coal seam top coal caving

**DOI:** 10.1038/s41598-025-16019-z

**Published:** 2025-10-01

**Authors:** Luchao Ju, Zhiqiang Wang, Liang Chen, Yang Wang, Zhiheng Cheng

**Affiliations:** 1 School of Energy and Mining Engineering, China University of Mining Technology-Beijing, Beijing, 100083 China; 2 School of Mining Safety, North China Institute of Science and Technology, Beijing, 101601 China

**Keywords:** Top coal caving, Thick coal seam, Numerical simulation, Stress distribution, Residual coal, Coal, Engineering

## Abstract

Given the lack of systematic research on the movement of overburden strata and damage characteristics of floor residual coal in the process of thick coal seam top coal caving, this study takes the Anping Coal Mine as the engineering background. The spatiotemporal evolution of overburden collapse, force chain transmission, and floor stress redistribution was investigated via the FLAC^3D^‒PFC coupled numerical method. Owing to the large mining space of the thick coal seam, the collapse of the roof has the progressive characteristic of “first two ends, then the middle”, with a measured collapse angle of approximately 57°, forming an evident caving band and residual coal accumulation zones. Moreover, the force chain network in the goaf evolves from sparse to dense, gradually forming a stable load-bearing structure. In addition, the porosity of the floor fluctuates from a wide range (0.15–0.43) to a more stabilized level (0.18–0.40) after compaction. Correspondingly, the vertical stress in the center of the residual coal floor increases from nearly 0 MPa to 1–3 MPa as the overburden load is transferred downward. The research results have important reference value for the destabilization mechanism of surrounding rock in thick coal seam mined-out areas and the design of residual coal remining and provide feasible ideas for improving mine productivity and guaranteeing safe production and the sustainable development of coal resources.

## Introduction

China’s energy endowment is coal rich, oil poor, and gas poor, and its coal production and consumption rank among the highest globally^1–5^. In the next decade, the pattern of China’s primary energy production and consumption dominated by coal will largely remain unchanged^6–10^. Therefore, fully developing and rationally utilizing coal resources, improving the recovery rate, and ensuring sustainable mining development are urgent challenges for China’s coal industry^11–16^. Comprehensive thick coal seam mining is a key method for high-yield coal mines in China and is crucial for utilizing coal resources and enhancing production efficiency^17–20^. Combined with geological and mining technology conditions, improving the mining recovery rate of China’s mines depends on both advancing technology and equipment and recovering residual coal left underground^21–23^. This approach optimizes ecological governance and underground space use and offers a new approach to mining thick coal seams in China^24,25^. Among China’s coal mining resources, thick coal seam reserves and production account for approximately 44%. China’s thick coal seam mining is dominated by top coal caving, resulting in 7–12% coal loss in the mined-out area, with an additional 4% loss at the end of the face and roadway. The thickness of residual coal in the single mined-out area is the greatest at the two ends, and the amount of coal lost in the middle is relatively small, but the coal is gradually compacted. Moreover, during thick coal caving, overburden rock collapses extensively, with complex interactions between roof movement and waste rock filling, leading to nonlinearity and uncertainty in surrounding rock evolution^26–28^. This not only affects the safe mining of the working face but also creates challenges for subsequent residual coal remining. Therefore, it is of great theoretical value and engineering importance to study the stress evolution law of the surrounding rock in the mined-out area during top coal caving of thick coal seams.

Scholars have studied the mechanical properties of rock by elucidating the stress distribution characteristics of coal seam mining through mechanical experiments^29–34^. For example, Asadi et al.^35^ investigated the dynamic tensile strength of rock samples with different defect lengths. Furthermore, the bonded particle model was used to simulate the grain size effect in different loading states^36^. These studies provide complementary perspectives on stress redistribution and failure mechanisms and provide useful references for analyzing the stress evolution of overburden rock under mining action^37,38^. On the other hand, scholars have investigated the stress evolution characteristics during coal seam mining through large-scale experiments or numerical simulations^39–43^. For example, Wang et al.^44^ studied the influence of Tian’an Shenghua Coal Industry’s residual coal roof on the coal wall ganging, end face leakage, and bracket load of the remining face and (developed) prevention methods. Roadway excavation through the intact empty area and remining of the working face via the surrounding rock stress distribution law for the Guanling Mountain Coal Mine were carried out, and the roadway location and support technology, the working face over the coal pillar and the empty channel when the bracket working resistance calculation method was used were obtained^45^. Moreover, to address the old residual coal remaining from the uncontrolled development of small coal mines, a “U-beam + grouting + letting pressure anchor” joint support system was constructed^46^. The use of filling and grouting technology to treat empty roadways was proposed to ensure the safe and smooth passage of the remining face^47^. Furthermore, Liu et al.^48^ invented an overrun support device for the overmining of broken perimeter rock in the remaining 3 m of the bottom coal remining face of the Taiping Coal Mine as the background. Feng et al.^49^ proposed key layer stacking and the breakage distance to address the mining of abandoned coal seams above the knife pillar mined-out area and developed the “5-step method” to determine the feasibility of upstream mining of abandoned coal seams above the knife pillar mined-out area. Taken together, more research has been conducted on the repeated mining of residual coal. However, the method still needs to be improved. In addition, the collapse and mechanical response of overburden strata after mining thick coal seams, including the damage characteristics of residual coal on the floor of the mined-out area and the vertical stress distribution law, must be studied. These studies would provide valuable databases for subsequent repeated mining of residual coal.

Although numerous studies have investigated overburden movement and residual coal recovery under various geological conditions, several key limitations remain. First, most previous studies relied on either continuum-based models or laboratory-scale experiments, which often oversimplify the complex interaction between roof collapse, waste rock compaction, and stress transfer in thick coal seams. These approaches usually neglect the granular mechanical behavior of collapsed materials and the stress transmission pathways through broken rock mass. Second, many models treat the goaf as a uniform void or rigid fill, overlooking the time-dependent evolution of force chains and porosity in the fill material, which is critical for understanding the feasibility of residual coal mining. Furthermore, existing studies often lack a detailed spatial-temporal coupling analysis of floor damage and stress redistribution under top coal caving conditions. Therefore, a more refined, multiscale approach is needed to capture the progressive failure and compaction behaviors in mined-out areas. To address these gaps, this study adopts a coupled FLAC^3D^–PFC modeling strategy to investigate the collapse evolution of overburden strata, force chain development, floor porosity variation, and stress redistribution during thick coal seam top coal caving.

This study focuses on analyzing the collapse characteristics of the overburden strata in the mined-out area, the distribution law of the force chain, damage characteristics of the coal‒rock interface, and the evolution law of floor damage and stress redistribution characteristics by constructing a numerical model. The results of this study can provide a reference for the management of surrounding rock in the comprehensive working face of thick coal seams and have certain significance for guiding the recovery of residual coal in mined-out areas.

## Methods

### Engineering background

The Anping Coal Mine, located in China, serves as the engineering background for this study. The mine is developed with inclined shafts and adopts the longwall mining method. The main coal seam currently being mined is the No. 5 − 1 seam, with a thickness of 9.2–14.2 m (average 11.7 m), and lies within the Taiyuan Formation. The roof comprises coarse sandstone and mudstone, while the floor consists mainly of mudstone and kaolinitic mudstone.

The 8118 working face of the No. 5 − 1 seam is 510 m long (strike) and 200 m wide (dip), with an average burial depth of approximately 188.72 m. The mining method involves full-mechanized top coal caving, with a mining height of 3.5 m and a top coal release height of 8.2 m. The caving process follows the “one cut, one release” principle and is managed by natural roof collapse. To ensure roof stability, top coal is not released near the head and tail ends.

The layout includes intake and return airways (each about 515–540 m long), with roadway cross-sections ranging from 4.5 to 5.5 m wide and 3.5 m high. The site is covered with loess and has minimal surface structures, ensuring favorable conditions for mining and simulation studies.

### Numerical modeling and model parameters

**Fig. 1:**
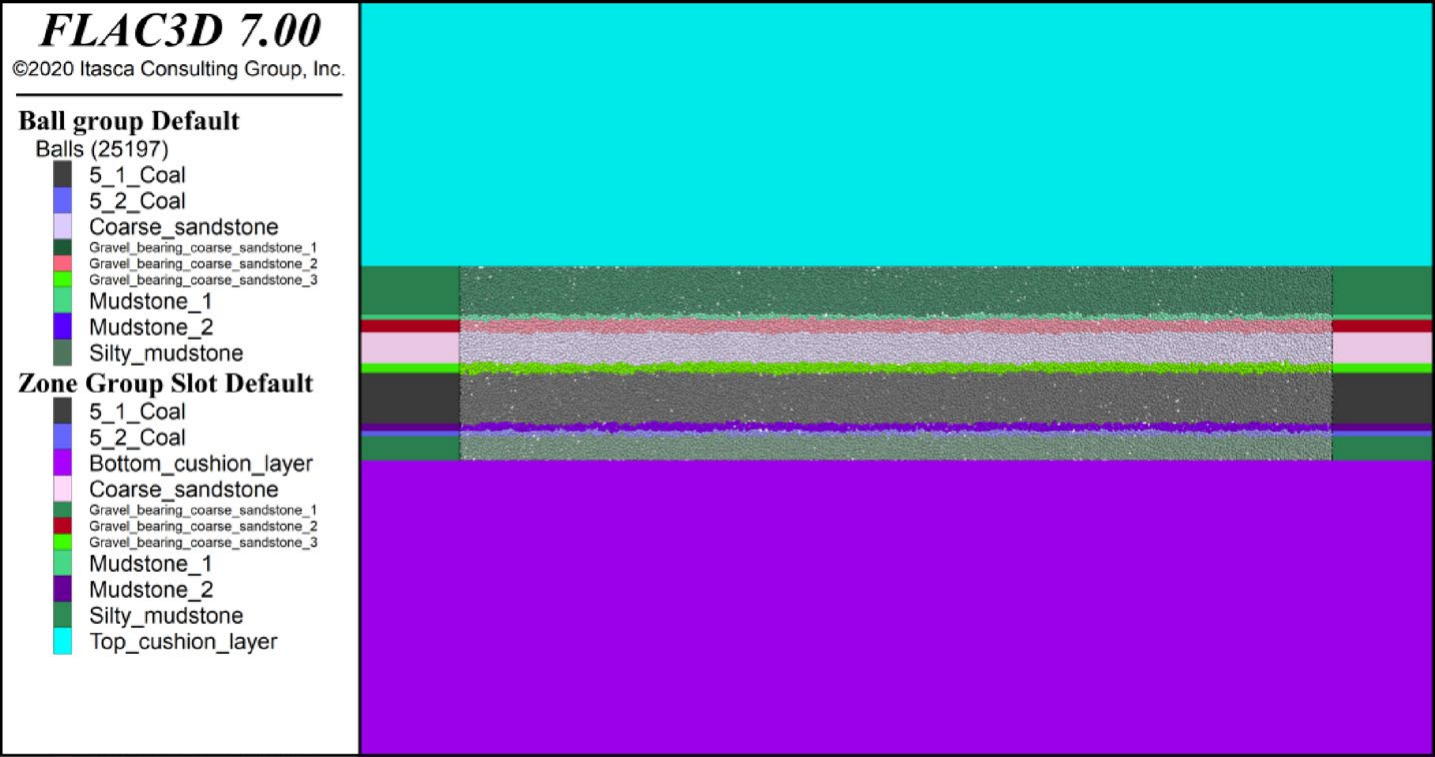
Numerical model of the 8118 working face.

Numerical analysis of the stability of the surrounding rock in the mined-out area is typically achieved via FLAC^3D^ finite-difference numerical simulation software, which sets the mined-out area as a null model and neglects the bearing effect of the collapsed waste rock on the roof overburden strata and the force transmission effect on the floor. However, the PFC particle discrete numerical simulation method can replicate multiple mechanical response characteristics of rock materials. Therefore, this study employs the FLAC^3D^−PFC coupled numerical model in FLAC version 7.0 to simulate the stress distribution and damage characteristics of the working face. The numerical model is shown in Fig. 1, with a model size of 1000 m × 397.12 m and a total of 11 layers: 6 roof rock layers, 1 main coal layer, and 4 floor rock layers. The middle part of the model is a PFC model that is 200 m long and covers the 9 layers in the middle, and 25,097 particles of different sizes are generated inside the model according to different lithologies. The left, right, and lower boundaries of the model are fixed, whereas the top is a free boundary. The average burial depth of the 8118 working face is 188.72 m, and the center of the working face is 100 m from the top of the model. The vertical stress to be applied is calculated to be 3.82 MPa.

**Fig. 2:**
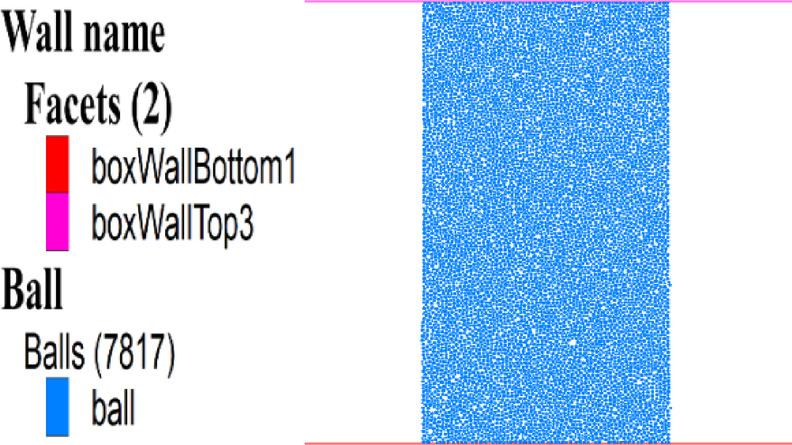
Model for calibrating the mesoscopic parameters.

To obtain the model’s mesoscopic parameters for the mining field, a two-dimensional mesoscopic parameter calibration model with the same scale as the PFC part in the coupled FLAC^3D^−PFC numerical model for the 8118 working face was created. The calibration model size is 50 m × 100 m, as shown in Fig. 2.

**Fig. 3:**
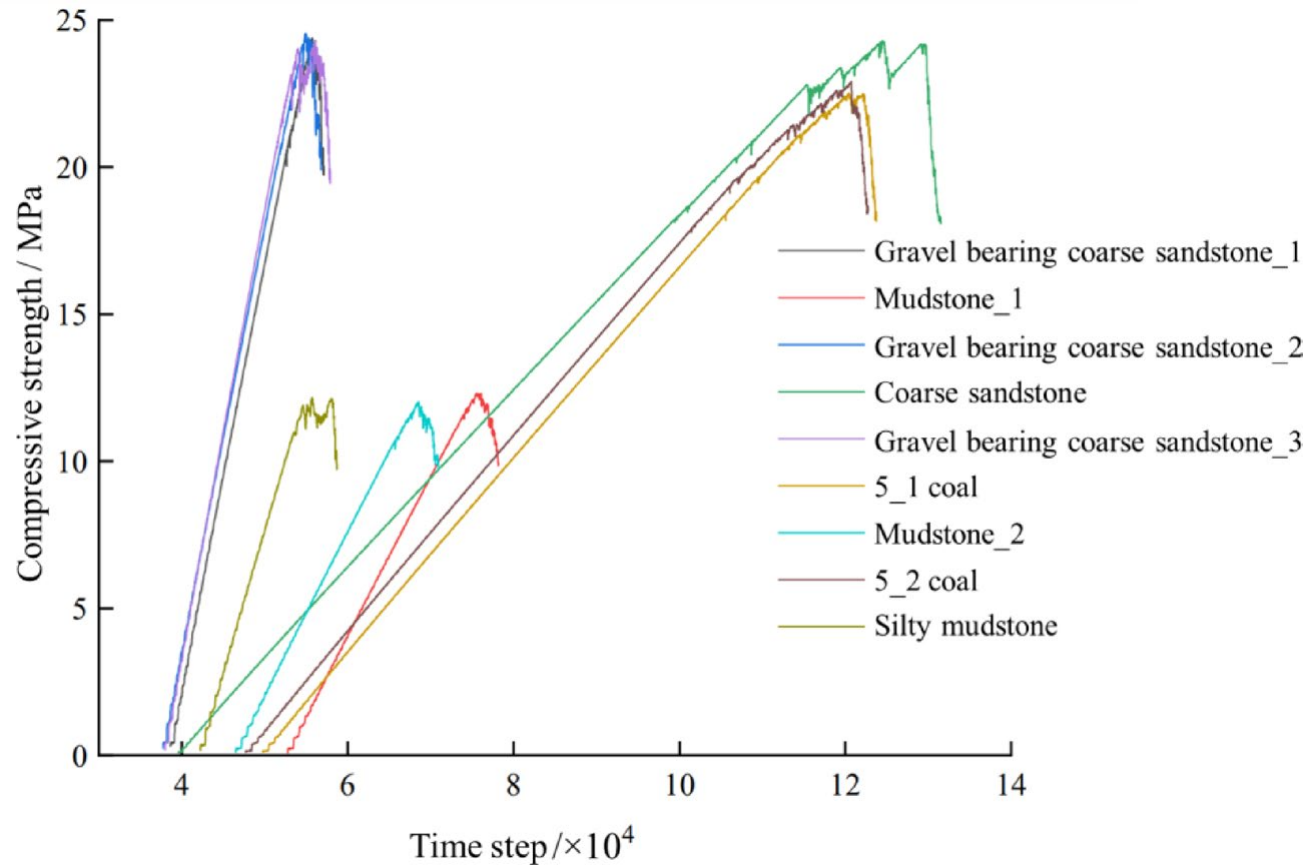
Stress‒strain curves of each rock layer in the 8118 working face.

Combined with the geological exploration report for the mine and literature^50–52^the physical−mechanical parameters and mesoscopic parameters of each rock layer at the roof and floor of working face 8118 in the Anping Coal Mine are shown in Tables 1 and 2. The mesoscopic parameters of each rock layer were then substituted into PFC software for a uniaxial compression test, and the stress‒strain curves for each rock layer were obtained, as shown in Fig. 3.

**Table 1:** FLAC physical‒mechanical parameters for each rock layer.

Name of rock layer	Density (kg/m^3^)	Thickness (m)	Bulk modulus (GPa)	Shear modulus (GPa)	Cohesion (MPa)	Internal friction angle (°)	Tensile strength (MPa)
Gravel bearing coarse sandstone_1	2540	11.15	19.05	7.59	7.43	41.4	1.3
Mudstone_1	2510	1.15	3.91	2.76	12.77	30.4	0.55
Gravel bearing coarse sandstone_2	2560	2.95	19.05	7.59	7.43	41.4	1.3
Coarse sandstone	2580	7.05	29.07	9.57	7.43	41.4	2
Gravel bearing coarse sandstone_3	2550	2.12	19.05	7.59	7.43	41.4	1.3
5_1 coal seam	1430	11.70	3.84	1.72	5.94	31.8	0.5
Mudstone_2	2530	1.65	3.91	2.76	12.77	30.4	0.55
5_1 coal seam	1450	1.15	3.84	1.72	5.94	31.8	0.5
Silty mudstone	2540	5.60	8.23	5.81	12.86	30.4	0.75

**Table 2:** PFC mesoscopic parameters of each rock layer.

Name of the rock layer	Minimum particle size (m)	Parallel bonding modulus (GPa)	Parallel bonded stiffness ratio	Parallel bonding cohesion (MPa)	Parallel bonding tensile strength (MPa)	Friction factors	Porosity (%)
Gravel bearing coarse sandstone_1	0.420	25.96	1.77	21.67	26.00	0.5	0.2
Mudstone_1	0.300	6.99	1.37	10.21	12.25	0.5	0.2
Gravel bearing coarse sandstone_2	0.420	25.73	1.77	22.13	26.56	0.5	0.2
Coarse sandstone	0.450	34.08	1.91	24.18	29.02	0.5	0.2
Gravel bearing coarse sandstone_3	0.420	27.69	1.77	21.39	25.67	0.5	0.2
5_1 coal seam	0.250	5.52	1.96	17.26	20.71	0.5	0.2
Mudstone_2	0.300	6.92	1.37	9.41	11.29	0.5	0.2
5_1 coal seam	0.250	5.49	1.96	16.94	20.33	0.5	0.2
Silty mudstone	0.350	15.59	1.39	10.51	12.61	0.5	0.2

### Simulation test program

**Fig. 4:**
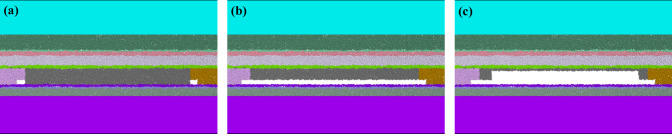
Simulation of the excavation process. (**a**) Excavation of the intake airflow roadway and return laneway; (**b**) excavation of the working face; and (**c**) top coal caving.

In accordance with the actual construction process for the 8118 working face, excavation was simulated in 3 steps. In the first step, the intake airflow roadway and return laneway of the 8118 working face were excavated in the designed position. The section size of the intake airflow roadway is 5.5 m × 3.5 m, and the section size of the return laneway is 4.5 m × 3.5 m. The second step is to excavate the 8118 working face after calculating equilibrium, with the working face having a section size of 100 m × 3.5 m. The third step is to release the top coal, with the height of coal release being 8.2 m until equilibrium is reached. The equilibrium condition is set such that the model limit average convergence ratio is 1 × 10^− 4^. The overburden strata stress distribution laws in the process of roof collapse, waste rock accumulation and compaction are analyzed and studied. The excavation steps are shown in Fig. 4.

## Results and discussion

### Characteristics of overburden strata collapse in mined-out areas

**Fig. 5:**
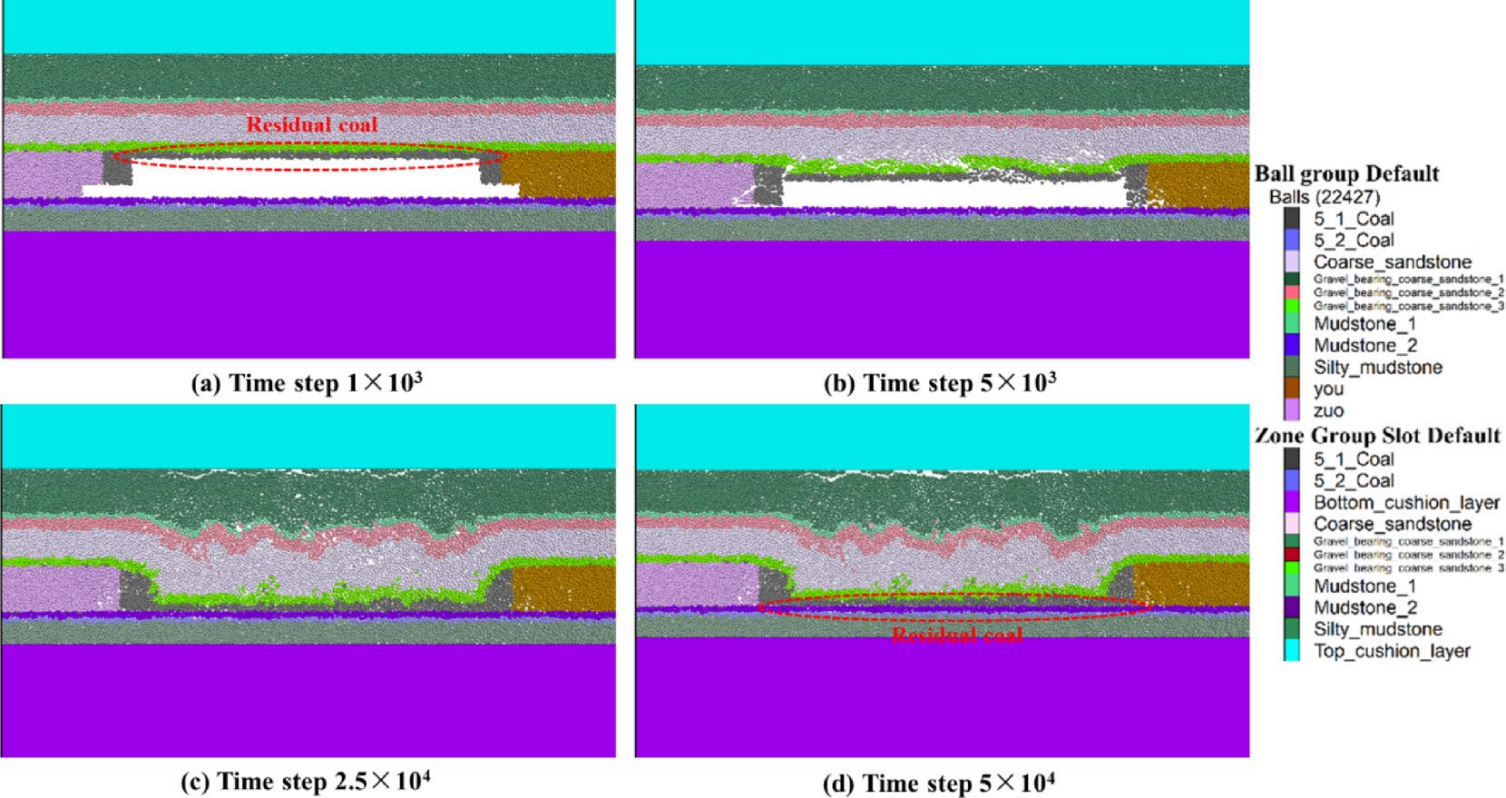
Characteristics of overburden strata collapse in the mined-out area after the top coal caving of thick coal seams. (**a**) Time step of 1 × 10^3^; (**b**) time step of 5 × 10^3^; (**c**) time step of 2.5 × 10^4^; and (**d**) time step of 5 × 10^4^.

As shown in Fig. 5a, coal seam excavation was carried out at a time step of 1 × 10^3^. As the time step increased to 5 × 10^3^, initial fractures appeared at both ends of the working face because of the high self-weight, whereas the central roof exhibited less rotation and subsidence, as shown in Fig. 5b. With increasing time step, the roof and overlying surrounding rock further fractured and collapsed into the mined-out area (Fig. 5c). Driven by self-gravity and surrounding rock pressure, the roof experienced progressive block collapse, with a marked increase in rotary subsidence. In the process of collapse, the rock layer of the roof may sequentially experience delamination, fissure expansion and falling blocks, and the overall performance is characterized by damage that gradually extends from the two ends to the middle. Because the coal seam is thick, there is enough space in the mined-out area after top coal caving; thus, top coal caving and rock block filling occur to a certain degree, which gradually reveals the environment necessary for the repeated mining of residual coal. As shown in Fig. 5d, the model reached equilibrium at a time step of 5 × 10⁴, illustrating the postmining collapse pattern of the overburden strata. At this stage, the rock mass in the mined-out area has sufficiently collapsed. Moreover, the top and two sides of the working face are in a state of relative collapse, and the broken rock and residual coal in the middle collapse area are squeezed and accumulate, forming a stable space for bubbling. In addition, the residual coal accumulates in the fallout area, forming a potential storage pattern for the repeated mining of residual coal, which provides a possibility for the subsequent recovery of residual coal.

In summary, owing to the large space of the thick coal seam during top coal caving, the fracture of the roof is not a one-time occurrence but rather occurs first from the two ends of failure and then expands to the center. In addition, the roof does not simply move downward in the process of collapsing but is accompanied by a certain degree of rotation and tilting rupture, which is more obvious, especially when the mined-out area is wider. The extensive mined-out area leads to a significant caving band in the overburden strata, and the caving blocks pile up and fill each other in the mined-out area. After collapsing, the working face retains part of the coal, and a certain scale of residual coal distribution is revealed in the accumulation of fallout and fractured coal, which provides the possibility of subsequent repeated coal mining.

### Distribution pattern of the force chain in the mined-out areas

**Fig. 6:**
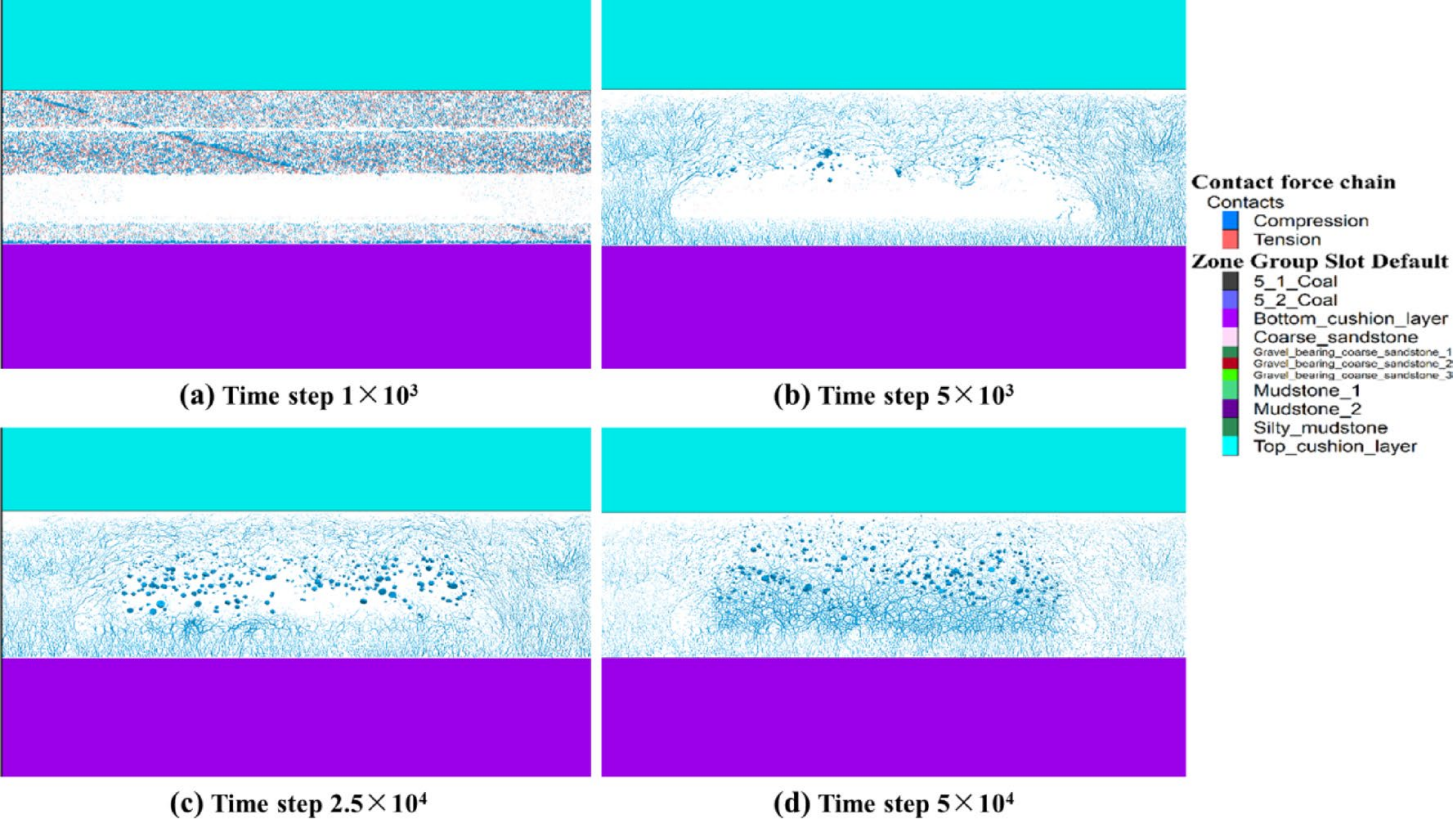
Characteristics of overburden strata force chain evolution after top coal caving of a thick coal seam. (**a**) Time step of 1 × 10^3^; (**b**) time step of 5 × 10^3^; (**c**) time step of 2.5 × 10^4^; and (**d**) time step of 5 × 10^4^.

The PFC model characterizes the distribution of contact forces between particles using force chains; their sparseness and color depth reflect the magnitude of stress, whereas their extension indicates stress transfer. Figure 6 shows the whole process of contact, pressure and re-establishment of bearing between waste rock particles and key blocks of overburden strata in the mined-out area.

Since the key block has not yet exerted significant compressive pressure on the waste rock, the floor of the mined-out area is subjected mainly to the self-weight of the waste rock, and the force chain is sparse, as shown in Fig. 6a and b. Because the key block on the waste rock is not yet extrusion pressure, the floor of the mined-out area in the vertical direction is subjected mainly to the gravity of the waste rock, and the force chain between the waste rock contact force chains presents a point or line distribution, which is quite sparse overall. This finding indicates that the compressive stress distribution is not continuous and that the bearing system has not yet formed. As shown in Fig. 6c and d, with the continuous accumulation of waste rock in the mined-out area and the gradual downward movement of the overburden strata, the waste rock particles were further extruded and contacted, forming a more compact force chain network. The force chains inside the waste rock begin to connect with each other and gradually pass to the key block at a higher position, resulting in continuous densification of the blue force chain distribution, as shown in Fig. 6. At this time, the contact range between the waste rock and overburden strata expands, the waste rock particles support each other and bear part of the weight of the overburden strata, the force chain develops from the lower part of the mined-out area to the upper part of the mined-out area, and the color shading indicates a gradual increase in the strength of the force chain. The spatial and temporal distributions and changes in the force chain show the paths of stress transfer and rock deformation development in the mined-out area.

Overall, the force chain diagrams reveal that from initial excavation to late stabilization, the contact between waste rock and key blocks in the mined-out area becomes progressively stronger, and the force chain between the particles changes from sparse to dense, which indicates that the waste rock pile is gradually compacted, bears the load of overburden rock, and forms a relatively stable mechanical support system. This force chain evolution law closely corresponds with roof movement, mined-out area fallout and the residual coal storage pattern in the top coal caving of thick coal seams.

### Characteristics of coal rock damage in the mined-out area

**Fig. 7:**
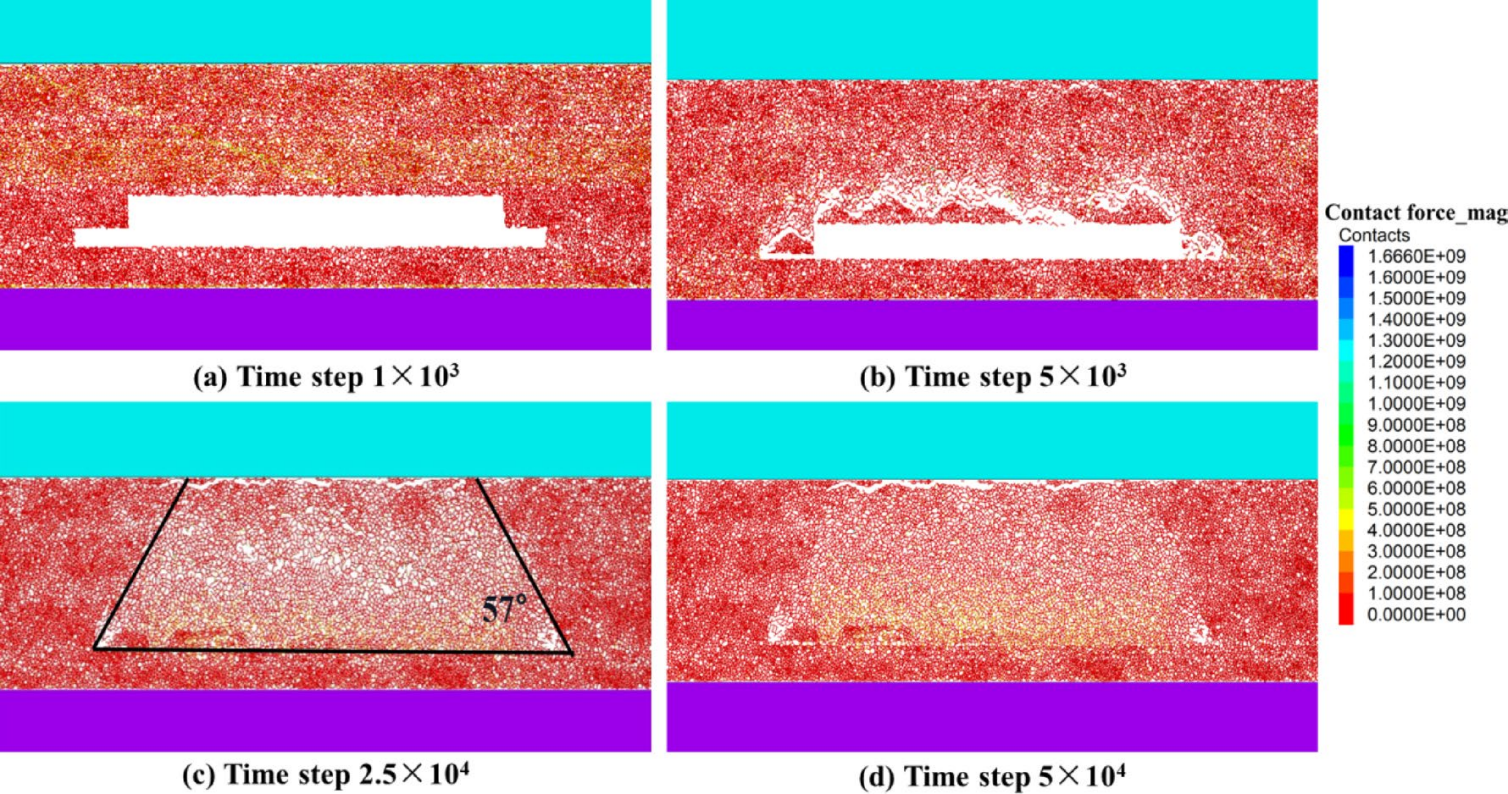
Characteristics of coal rock destruction in the mined-out area after top coal caving of a thick coal seam. (**a**) Time step of 1 × 10^3^; (**b**) time step of 5 × 10^3^; (**c**) time step of 2.5 × 10^4^; (**d**) and time step of 5 × 10^4^.

After the 8118 comprehensive workface is mined, the parallel bond between the coal particles is first broken under ground stress in the unplaced coal section at the end of both sides of the mined-out area, causing shear slip damage along the hollowed coal seam; second, the solid coal becomes the main carrier of the overburden strata loads, and after the coal along the hollow area collapses, the roof overhanging area increases in size, and the loads increase accordingly. The roof gradually breaks up and crumbles under greater loads, as shown in Fig. 7a and b. As shown in Fig. 7c, as the roof continues to sink, the stress level is gradually transferred to the overburden strata, causing shear and rupture in the coal or surrounding rock at relatively high levels. Eventually, a trapezoidal failure profile formed above the mined-out area, indicated by the black lines in Fig. 7c, and the collapse angle on both sides was measured to be approximately 57°. Moreover, due to the release of top coal, many broken waste rocks fell from the roof to the floor of the mined-out area, where they gradually accumulated and compacted. The parallel bonds between the coal gangue particles and the floor rock particles reconnected under extrusion, and the supporting effect was partially restored. This filling from the bottom to the top causes the failure zone of the mined-out area to have a relatively symmetrical outline, which is favorable for the overburden strata to reach a new equilibrium state to some extent. As shown in Fig. 7d, when the roof continued to sink and crumbled, the crumbled rock and coal mass filled the mined-out area. The filling body was gradually compacted by pressure. The mined-out area eventually formed a left−right symmetrical avalanche accumulation pattern, and the contact force between the roof and waste rock was relatively balanced. This symmetry is attributed to relatively uniform geological conditions on both sides of the working face. This is shown by the color distribution of the contact force between the upper part of the mined-out area and the floor. The high-stress zone gradually shifts upward with the closure of the collapse space and eventually forms stress arches or broken arches at the top and both sides with a certain pattern.

In summary, the simulation results show that in the process of mining thick coal seams, the destruction of coal and rock is not a one-time overall occurrence but follows the progressive “first side and then middle, first lower and then upper” law of collapse. This process is affected by the joint influence of ground stress, roof structure and waste rock filling characteristics and ultimately results in a stable symmetrical collapse profile. The measured angle of collapse on both sides is approximately 57°. The gradual collapse and filling of the mined-out area redistribute the stresses in the overburden strata in space. The two sides are subject to concentrated loads and fail first, whereas the center becomes the main loaded area at a later stage.

### Damage evolution law of the floor in the mined-out area

**Fig. 8:**
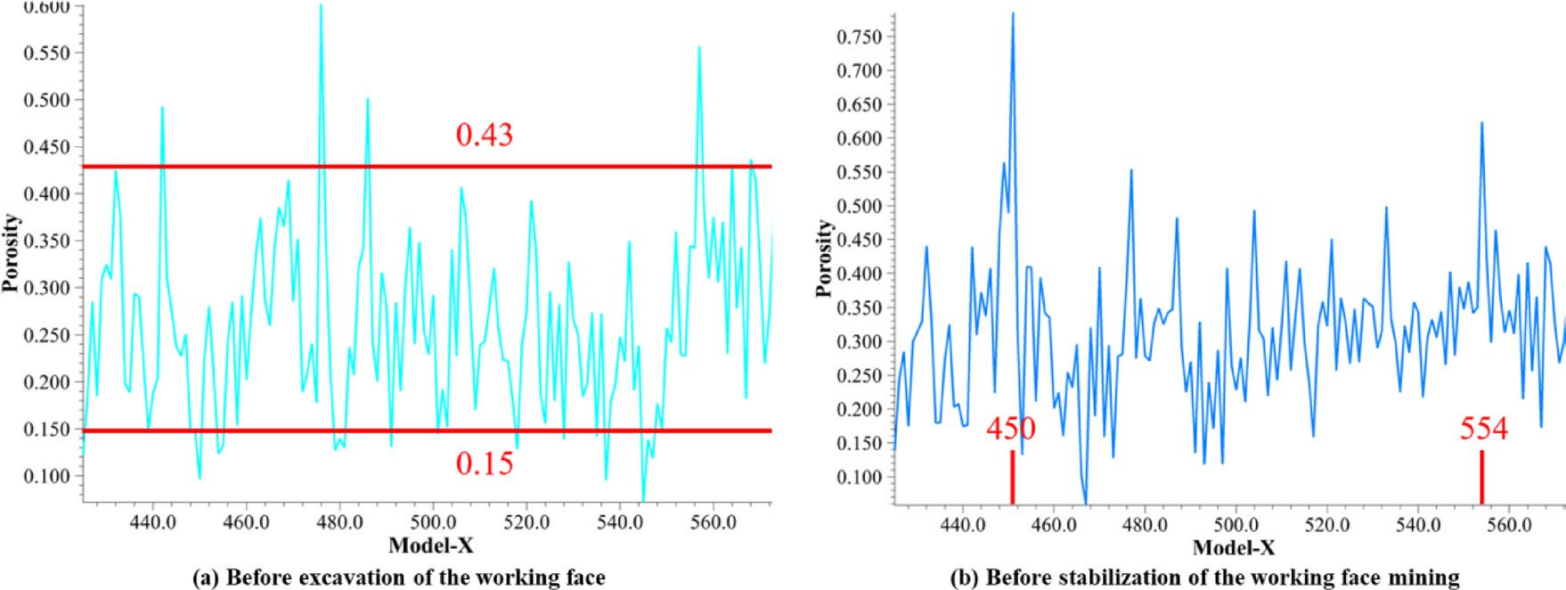
Porosity of the floor of the mined-out area. (**a**) Before excavation of the working face; (**b**) before stabilization of the working face.

The porosity at the floor of the 8118 mined-out area was monitored using a measuring circle, allowing the evolution pattern of floor porosity to be determined. Before the working face is excavated, the porosity of the floor of the mined-out area is relatively stable, as shown in Fig. 8a. In the unexcavated stage, the floor of the working face has not been disturbed by mining, and the coal mainly bears the self-weight of the original rock and the lateral stress of the surrounding rock. The porosity is distributed in the interval of 0.15 ~ 0.43, and although the curve fluctuates to a certain extent, the amplitude is limited.

As shown in Fig. 8b, when the working is mined but has not yet fully stabilized, there are obvious high porosity peaks near X = 450 m and X = 554 m on the measurement line. Combined with the above, the two locations are exactly at the junction of the intake airflow roadway and return laneway and the section in which no coal release occurs. As the low key layer cuts the edge of the mined-out area, the high key layer forms an articulated structure at the edge of the mined-out area, and there are 4 racks (7 m) of unreleased top coal on the right side of the end and 3 racks (5.25 m) on the left side of the end, which collapse and accumulate at the edge of the mined-out area, supporting the direct top. Thus, the location of the basic top fracture is biased toward the mined-out area, with a certain distance from the coal wall, which forms the separation zone. This causes the coal to be insufficiently compacted, resulting in a localized porosity that is higher than that in other areas.

**Fig. 9:**
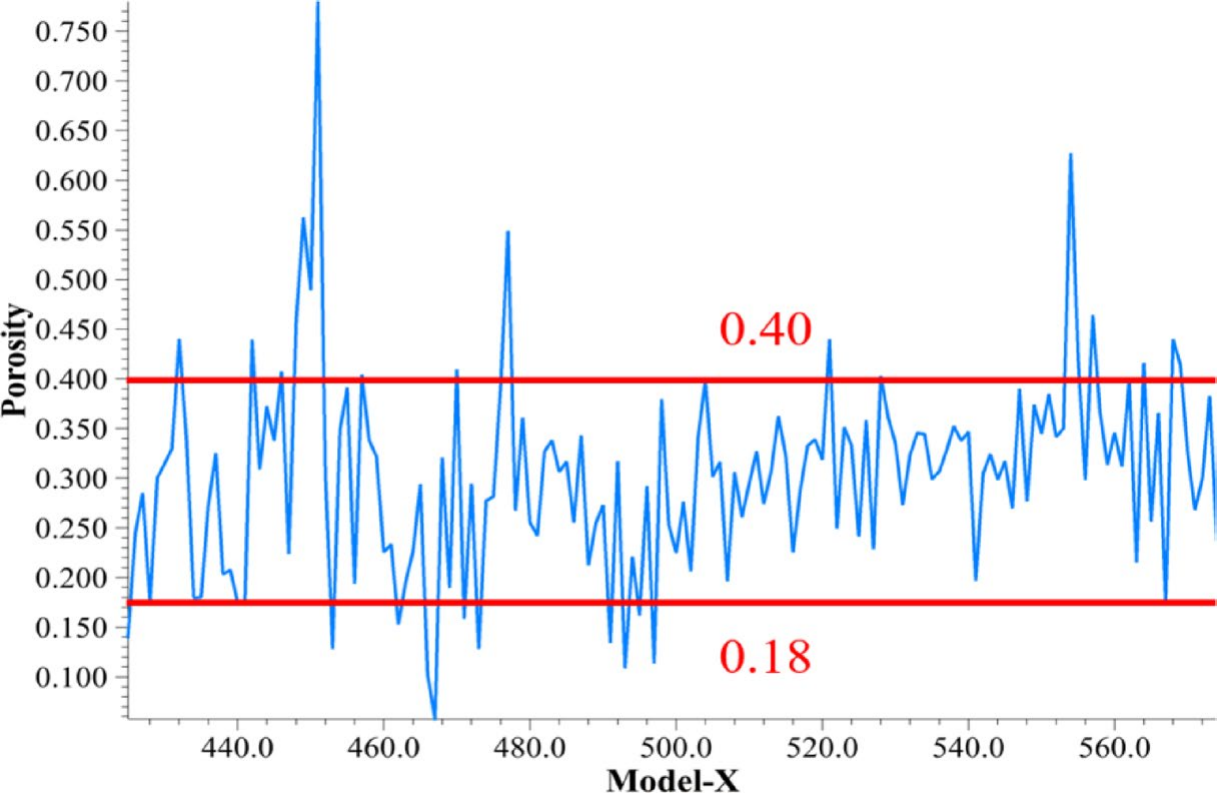
Porosity of the floor of the mined-out area after stabilization of the working face mining.

As shown in Fig. 9, after the working face enters the mining stabilization stage, the roof of the mined-out area has basically collapsed, the waste rock has fully accumulated, and the residual coal of the floor is further compacted under continuous stress, with porosity mostly concentrated in the narrower range of 0.18 to 0.40. With the advancement of mining, the residual coal and waste rock continue to squeeze down and fill the floor so that the pore structure is relatively stable, indicating that the residual coal of the floor in the new stress equilibrium environment no longer has a large-scale pore rate of high or low values.

Overall, porosity experienced wide fluctuations during early mining and localized high values in transitional phases and eventually stabilized as the degree of compaction increased. The dominant mechanism lies in the cutting down position of the key layer of the roof and the local supporting effect of the unremitting coal section, resulting in high-porosity zones at the edge, which are gradually compacted and stabilized as waste rock and top coal fill the space.

### Vertical stress distribution law of residual coal in the floor of the mined-out area

**Fig. 10:**
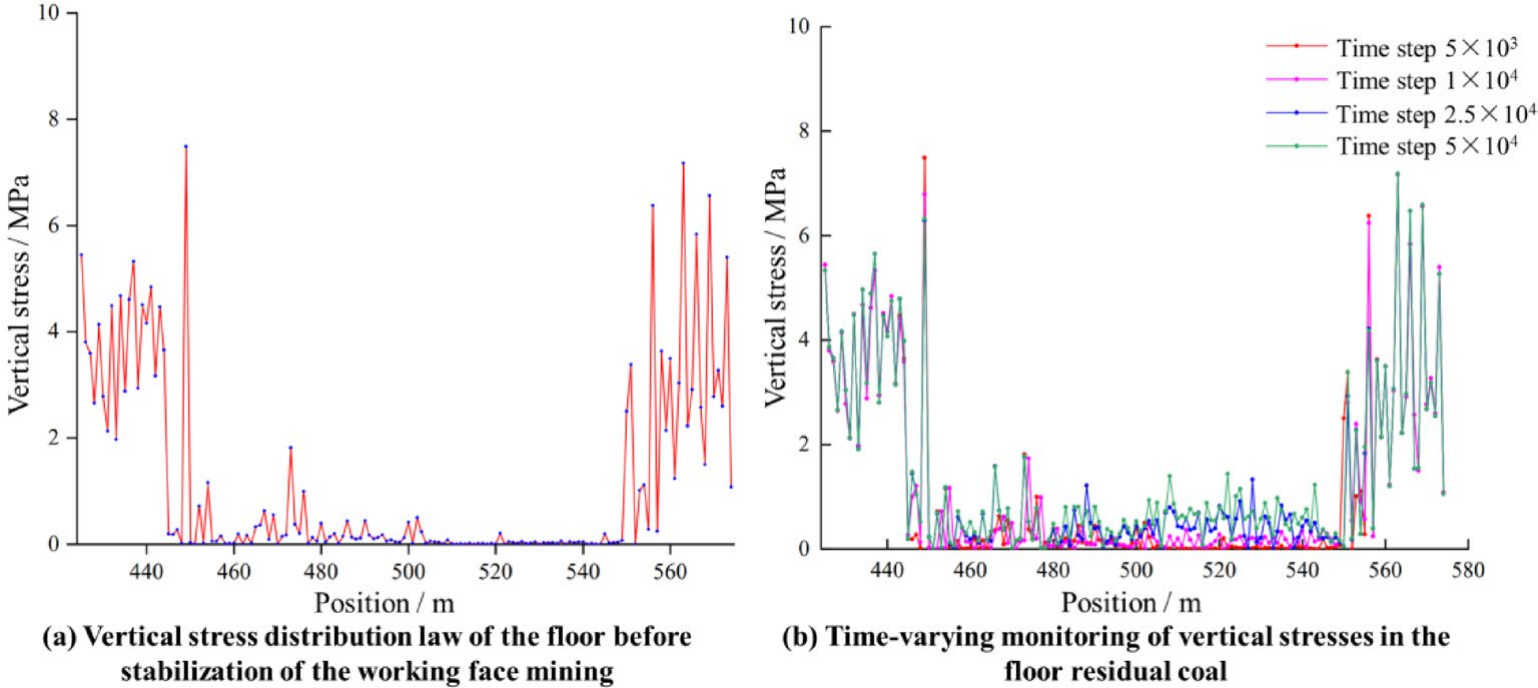
Distribution of the vertical stress. (**a**) Vertical stress distribution law of the floor before stabilization of the working face; (**b**) time-varying monitoring of vertical stresses in the floor residual coal.

By arranging the measuring circle at the floor of the working face, the vertical stress of the residual coal at the floor of the 8118 mined-out area was monitored, and a curve of the vertical stress of the residual coal with time was obtained. As shown in Fig. 10a, when the working face has not yet experience complete collapse or the fallout has not yet been compacted, the vertical stress in the middle part of the measurement line is close to 0 MPa, and only near the coal wall or the support side (e.g., near 440 m, near 560 m) does a small increase in stress occur. This is because the mined-out area has not been fully filled and compacted by the top coal or waste rock, and the overburden rock is essentially “suspended” or supported only at the edges. The floor in the center of the mined-out area almost does not bear the load, self-weight or roof gravity in this transfer path, or the two sides of the unmined coal wall bear weight; thus, the vertical stress measurement value is close to zero.

The distribution of vertical stress on the floor of the mined-out area at multiple time steps is given in Fig. 10b. With increasing time step, the vertical stress in the middle of the mined-out area, which was close to 0, began to increase gradually. This indicates that as top coal and waste rock accumulate and compact, the overburden load gradually transfers to the residual coal at the floor. At the same time, the original high stress on both sides spreads to the center of the mined-out area, resulting in the residual coal on the floor in the region also beginning to bear some stress. The mined-out area changed from “weakly supported” to “waste rock−residual coal”, and the vertical stresses at each location tended to increase.

**Fig. 11:**
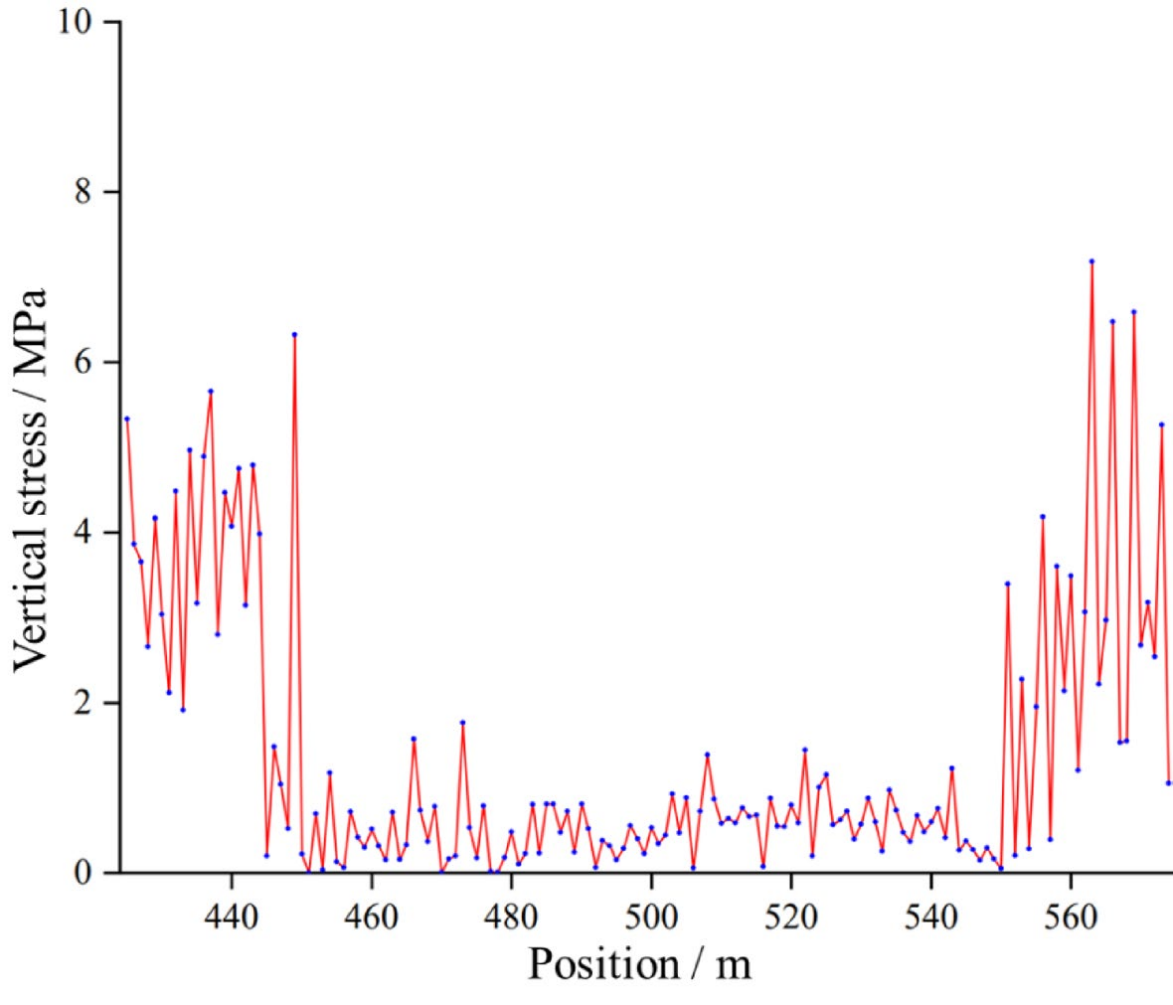
Vertical stress distribution law of the floor after stabilization of the working face.

As shown in Fig. 11, when the working face is mined into the stabilization stage, the vertical stress of the floor no longer fluctuates greatly, and a relatively clear stress gradient forms between the center and the sides. The stress on both sides is greater, generally up to 5–8 MPa; the stress in the center of the mined-out area is lower than that on the two sides, but it is no longer close to 0 and is usually maintained in the range of 1–3 MPa. This depends on the degree of compaction of the collapsed material. This is because the collapsed blocks from the roof, waste rock and residual coal in the mined-out area accumulate in dense areas and can be gradually established to overburden the rock load transfer to the floor of the bulk carrier. Moreover, as the central part of the mined-out area is filled with waste rock and residual coal, the overlying gravity is transferred to the residual coal of the floor by the falling body so that the stress in the area is restored to a certain level, resulting in a relatively uniform and stable distribution.

In summary, the change in vertical stress of the residual coal of the 8118 working face floor is revealed, reflecting the close coupling between the formation of the impudent fallout in the mined-out area and the stress redistribution of the floor in the top coal caving of the thick coal seam. When the roof of the working face has not collapsed completely, the two sides of the mined-out area are the first to carry high stress, whereas the middle part of the mined-out area is almost unloaded. With the filling of the fallout body and the formation of the load-bearing structure, the stress is transferred from the side to the middle part and finally tends to stabilize.

## Conclusions

This study employed a coupled FLAC^3D^–PFC numerical model to investigate the collapse evolution, force chain development, floor porosity, and stress redistribution in the mined-out area during top coal caving of thick coal seams. The following conclusions can be drawn:

1. Roof fracture during top coal caving of thick coal seams exhibits a progressive pattern of “first the ends, then the center,” accompanied by rotational subsidence. This leads to the accumulation of fractured coal and residual coal in the mined-out area, providing the physical basis for potential repeated mining. Concurrently, the force chain between waste rock and overburden key blocks evolves from sparse to dense, indicating a gradual transition to a stable load-bearing structure.
2. A symmetrical collapse profile is formed under the combined influence of in situ stress, roof structure, and waste rock compaction, with a measured collapse angle of approximately 57°. The stress redistribution follows the sequence of “ends first, center later,” ultimately forming a more uniform load-bearing state within the goaf.
3. The floor porosity decreases from the initial fluctuation (0.15–0.43) to a stabilized range (0.18–0.40) as mining progresses and compaction intensifies. Correspondingly, the vertical stress in the central residual coal floor gradually increases from nearly zero to 1–3 MPa, reflecting the upward transmission of the overburden load through the collapsed fill.

Limitations and Future research: Field-scale heterogeneity, groundwater influence, and gas pressure effects were not considered, which may influence actual floor damage and stress transfer mechanisms. Future studies should incorporate multi-physics coupling and field monitoring data to validate and refine the modeling framework. Moreover, the mechanical behavior of residual coal under secondary mining should be further examined to support safe and efficient re-mining strategies.

## Data Availability

All relevant data are within the manuscript.
